# The rice Trait Development Pipeline: a systematic framework guiding upstream research for impact in breeding, with examples from root biology

**DOI:** 10.1007/s11104-025-07399-2

**Published:** 2025-03-29

**Authors:** J. Damien Platten, Amelia Henry, Dmytro Chebotarov, Van Schepler-Luu, Joshua N. Cobb

**Affiliations:** 1 Rice Breeding Innovations Department, International Rice Research Institute, Los Baños, Laguna, 4031 Philippines; 2 Present Address: Global Breeding Transformation, RiceTec Inc, 1925 FM 2917, Alvin, Texas 77511 USA

**Keywords:** Rice, Root, Breeding, QTL, GWAS, Trait development, Population

## Abstract

**Background:**

In crop breeding, ‘trait development’ is the improvement of specific characteristics, typically using landraces as a source for introduction into elite lines. Trait development exists upstream of ‘breeding,’ which generates new varieties to be grown by farmers. While both are active areas of research, trait development is often overlooked, despite being a critical step in linking upstream research with breeding. The field of root biology provides many excellent examples of upstream research that requires further trait development to generate new varieties.

**Scope:**

Here, we describe the IRRI rice Trait Development Pipeline which provides a framework of clear protocols to discover, test and validate research outputs and maximize their potential for impact in mainstream breeding. We recommend specific steps in the context of further trait development for several rice root biology studies based on the guidelines established in the IRRI rice Trait Development Pipeline. Common trait development recommendations for areas such as root biology include ensuring the relevance of studied traits to field performance, rigorous testing to ensure reliability of genes and marker systems in elite backgrounds, and the packaging of those genes into elite material that can be easily used in breeding.

**Conclusion:**

In implementing the Trait Development Pipeline, it is expected that recurrent selection-based breeding strategies will benefit more from linkages with upstream research areas, such as root biology, by implementing marker-assisted selection to increase the frequency of large-effect rare alleles that currently exist outside the elite gene pool without hindering the genetic improvement that comes from quantitative breeding methods.

**Supplementary Information:**

The online version contains supplementary material available at 10.1007/s11104-025-07399-2.

## Introduction

Development of new, improved varieties of crops involves optimization and improvement of a wide variety of traits, including yield, quality, disease resistance, and stress tolerance. Research enabling this trait improvement is often captured under the umbrella of trait development, or pre-breeding, and includes trait discovery, trait validation, trait engineering, trait integration (of more than one trait), trait evaluation, and trait deployment and monitoring. By managing the introduction of novel large-effect alleles into elite backgrounds useful to breeding programs, trait development accelerates the breeding process by generating a step-change in the mean genetic merit of an elite line. In this manuscript we present the IRRI rice Trait Development Pipeline (the “Trait Development Pipeline”), a systematic, step-by-step process to identify, develop, and implement desirable traits in crops (Platten and Henry 2023), which aims to identify new sources of genetic variation for important traits, elucidate their genetic control, and make novel genetic variation available for easy use by breeders.

One area of research that stands to benefit from trait development is the linking of root biology with breeding. Root traits confer important adaptive advantages to many climate-related stresses that are affecting crop productivity. Given the difficulty of phenotyping root traits under conditions that are relevant to farmers (i.e. in soil, under field conditions), the improvement in our understanding of root biology in the previous decades has been remarkable. However, the level of adoption of root biology research outputs by crop improvement programs has been less than could be desired. Meanwhile, breeding programs are struggling to achieve the rates of genetic gain required to meet growing demand (Seck et al. 2023).

Trait discovery studies, including many root biology studies, typically involve screening of diversity panels, physiological dissection of tolerance/resistance mechanisms, and gene/QTL identification. While the majority of these studies involve manipulation by transgenics or genome editing, a significant number describe effects from naturally-occurring variation that could be applied in plant breeding in a straightforward manner. However success stories documenting the effective use of these genes in mainstream breeding (and subsequent deployment in farmers’ fields) are much less common (Khaipho-Burch et al. 2023). This lack of uptake limits the potential progress by breeding programs. Even where genes are known and characterized, many of these are not utilized in mainstream breeding (Platten et al. 2019; Wissuwa et al. 2016). This is particularly noticeable with disease resistance, where many of the most effective genes for blast and bacterial blight resistance are completely absent from mainstream breeding programs (Juma et al. 2021). In the absence of these genes, breeders are reduced to relying on phenotypic selection—competing with improvement in yield—and on genes of lower overall effectiveness.

Possible reasons that many traits/genes/QTLs that have been identified but are not getting used include a) lack of sufficient evidence for the reliability of the gene/QTL under field conditions, b) lack of available materials necessary for utilization (high-quality donors, reliable markers) and c) lack of awareness of the trait discovery research output by breeders. Understanding what factors are contributing to the lack of application in mainstream breeding is essential, and from this, a strategy to address these limiting elements can be devised to guide the trait development efforts.

## Key requirements of trait development for breeding purposes

In considering how to make trait development products, such as root traits and QTLs, more attractive to breeders, it is necessary to understand a modern breeding program (i.e. Bhosale, Thathapalli-Prakash et al. In review), and from there determine where the usefulness of trait development products is maximized. In backcross-based breeding programs, genes are often introgressed into existing popular varieties to improve those varieties one trait at a time with no intentional effort to generate genetic gain for quantitative characters. In this case, the ceiling for improvement remains the existing popular variety. However in forward breeding programs, the focus is on generating genetic gain for quantitative characters through the improvement of populations over multiple breeding cycles. At each cycle, superior segregants are identified, and selected individuals are recycled to use as parents for the next generation. These progeny form an “elite” pool of material through the accumulation of valuable polygenic haplotypes over many generations. To preserve the integrity of these elite haplotypic combinations, recurrent selection necessitates a closed pool of parents, drawn solely from the superior elite segregants of the previous cycles. Exotic material (such as from landraces and Genebank accessions) is not typically used directly in crosses to avoid disrupting the haplotypic combinations that have been meticulously assembled through generations of breeding. Consequently, some form of trait development is needed to link upstream work on Genebank accessions with elite breeding pools.

Qualitative genetic variation is controlled by one or a small number of loci (oligogenic) with major effects, such as is commonly seen in disease resistance traits. Most trait development activities aim to identify such variation. The intent of marker-assisted selection (MAS) is to replace (or at least supplement) existing phenotypic/polygenic selection processes for a certain trait (involving many small-effect loci) with oligogenic methods (few loci of significant effect). Breeders therefore need to be confident that marker-assisted selection will be effective by knowing that:

- the major loci (genes/QTLs) are reliable: they are being used to replace or supplement phenotypic or genomic selection for a particular trait. Reliability of a locus includes both effectiveness in the field, and in the germplasm a breeder is working with.
- the marker systems are reliable: selection will typically use low-cost marker-assisted technologies. The marker systems must accurately select for the desirable and undesirable alleles or haplotypes across the range of diversity encountered in the target breeding programs, and possibly much more widely.

To achieve this integration of oligo- and polygenic selection, trait development must therefore address the following issues:

- There should be no reasonable doubt the gene/QTL is conferring a useful benefit for relevant traits under field conditions.
- There should be adequate evidence of its effectiveness across a diverse range of elite genomic backgrounds.
- Selection methods should be reliable, high throughput and low cost.
- Validation (phenotyping) protocols must be available to breeding programs.
- The gene/QTL must be available in diverse, high-quality elite lines for use as parents in the breeding programs, without yield or genomic penalties from the original poor-quality donors.

The trait development process can subsequently be designed to specifically address these requirements.

## Structure and operation of the TDP

Trait development is a complex process, involving numerous activities and steps. To systematically organize this, a stage-gate system has been adopted. Stage-gate systems are a common framework used to formalize product development pipelines (Covarrubias-Pazaran et al. 2022), whereby the development process is divided into major stages, each representing significant milestones along the product development process. At the conclusion of activities in a stage, a review process is undergone against defined advancement criteria (the gate), at which point a decision is made whether to advance to the next stage. This process ensures major milestones are met, quality standards are enforced, and if problems and issues arise that mean further development is unlikely to be productive, that development terminates as soon as possible.

**Fig. 1:**
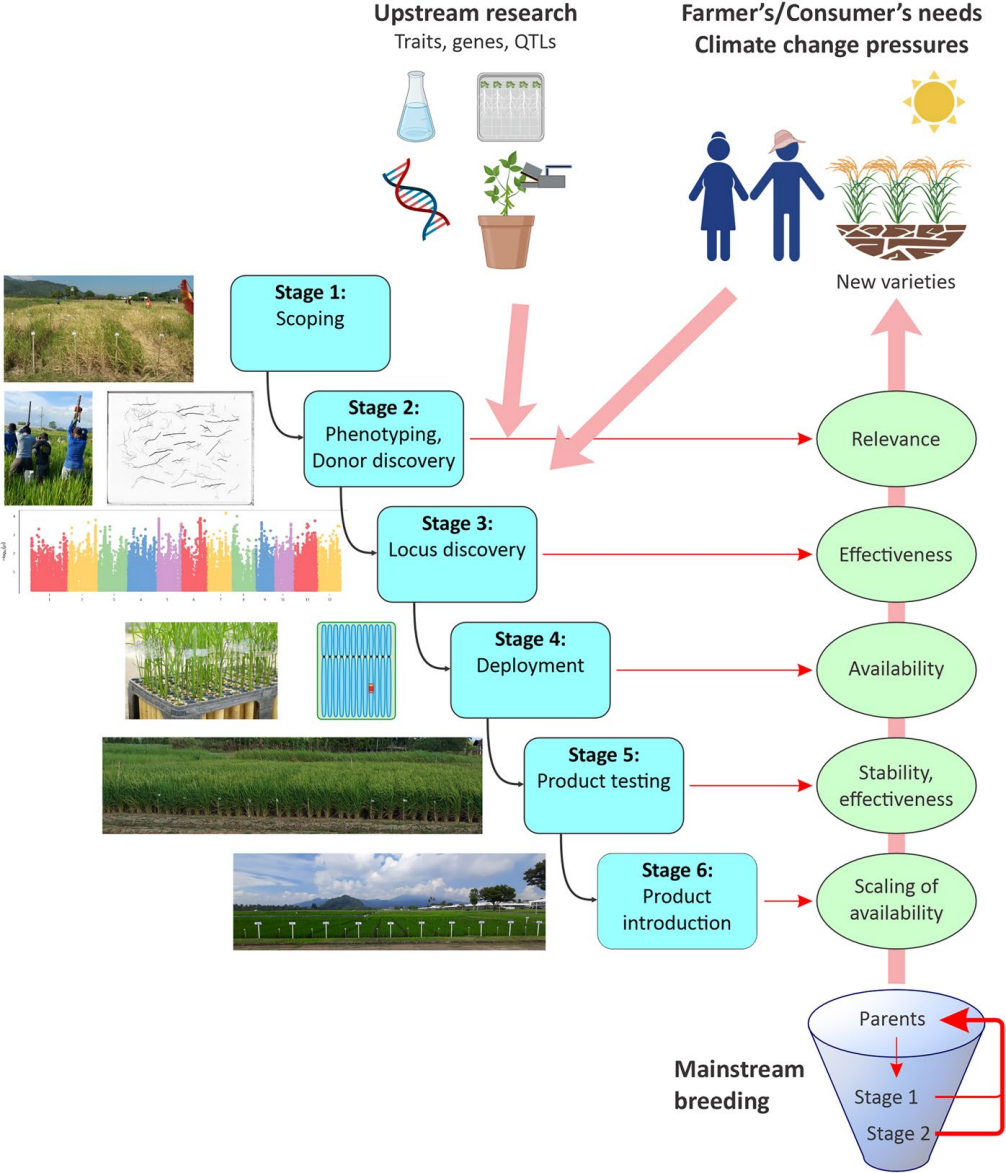
Overview of the Trait Development Pipeline process. Each stage is aimed at addressing one or more major issues required for confident application of MAS in mainstream breeding. The images illustrate an example implementation of the Trait Development Pipeline from a root/drought study (from top to bottom): stage1: field drought screening of a diversity panel with a range of phenotypes and durations among accessions, stage 2: phenotyping by root sampling in the field (left) and an example root scan from field samples (right), stage 3: Manhattan plot from GWAS on the diversity panel root phenotype results, stage 4: Deployment of promising root QTLs identified from the GWAS by introgression into an elite background, stage 5: field drought testing of deployment lines showing uniform phenotypes and durations among lines, stage 6: amplification and diversification of elite donor lines

In applying this paradigm to trait development in rice, a Trait Development Pipeline (TDP) structure was developed as shown in Fig. 1, with advancement criteria summarized in Table S1. This organizes trait development into six stages, beginning with evaluation of demand and proceeding through to delivery of materials and information to breeding programs. The structure entails an iterative nature focused on several cycles of evaluation: stage 1 evaluates whether trait development is needed, after which stages 2 and 3 are focused on initial discovery, during which evaluation is necessarily crude and preliminary. Promising loci are refined in stage 4, leading to a more rigorous validation in stage 5. Loci passing the stage 5 evaluation are then scaled up and final confirmation established in stage 6. Each validation cycle serves to give a greater level of detail and certainty regarding candidate loci, and hence multiple points at which development could be halted if it appears the locus is not suitable. Conducting such cycles of validation is important in providing the confidence in locus effect that is needed before advocating its use in breeding. The process is complex and will involve contributions from multiple expert teams. However the stage-gate framework and clear quality control targets outlined in the advancement criteria show each team what they need to produce to link with the next stage of development, and hence maximize the chance of advancement.

### Stage 1: project scoping

Stage 1 aims to evaluate whether trait development is required, to organize existing information about the trait, and formulate a trait development plan. Determination of whether trait development is needed is a function of demand (market-driven demand, where possible, and based on known gaps in the elite breeding material). This may be modified further based on relative trait priority and available resources. Assuming demand justifies investment in trait development, reviews of literature regarding known donor varieties, genes, screening protocols, marker systems etc. helps place prospective activities in relation to other work done. It is quite common, at least in rice and including root biology studies, to find previous studies for many traits that have investigated genetic variation contributing to phenotypic differences. In this case the known donors, genes and QTLs etc. can be evaluated against the same advancement criteria outlined in the TDP, thereby highlighting strengths and weaknesses in the current body of knowledge, whether it has achieved the major outputs required:

1. Has the gene been validated in relevant elite backgrounds?
2. Has the gene been validated under relevant field conditions or against relevant pathotypes?
3. Are donor materials available (elite or non-elite)?
4. Is the favorable allele already present in elite materials, particularly at high frequencies?

By evaluating literature against the predefined advancement criteria of the TDP (Table S1), strengths are noted and gaps are highlighted. Trait development activities can then be tailored to specifically fill these gaps. For example, although QTL mapping and even cloning studies have been carried out, validation in elite material is rarely conducted. Hence if a gene has been cloned and has reasonable evidence of efficacy, but is not available in accessible elite backgrounds, it may be possible to skip stages 2 and 3 and proceed directly to deployment of a cloned gene to elite backgrounds for validation. In another example, if a cloned gene is already at modest frequencies in current elite material, the only trait development work needed is the design and implementation of reliable marker systems. This gap analysis informed by the TDP then forms the basis of a trait development plan.

### Stage 2: screening protocol, donor discovery

In the event current knowledge is not sufficient to meet breeding targets, stage 2 is the initiation of actual research and development for the target trait, and is responsible for three critical outputs:

1. Development of a reliable, farmer-relevant screening protocol
2. Assessment of the variability for a trait in the current breeding pool
3. Discovery of donor material with substantially improved trait value, possibly combined with GWAS

Development of a screening protocol is one of the most critical outputs of the trait development process. This protocol will be used at every step of the way, from identifying donors and QTLs, validating introgression products and through to validating breeding products once the trait development process is complete. It is therefore essential that the screening protocol be relevant to actual field performance and that the screening focus on a field-relevant trait (rather than component traits), since they are how farmers assess performance. For example with stress tolerance traits, traits relevant to field performance may include maintenance of growth and/or health under stress at the vegetative-stage, while at reproductive stages this would likely be maintenance of yield under stress. Multiple physiological and genetic mechanisms (“component traits”) could contribute to these overarching field-relevant “composite traits”. For example, Fonta et al. (2022) considered shoot biomass as the composite trait in a study on vegetative stage drought stress and rice. They identified multiple “integrated root phenotypes” (Klein et al. 2020) – combinations of root traits that were related to the composite trait of shoot biomass. One integrated root phenotype was comprised of high nodal root number at depth, large root cross-sectional area, and a high proportion of root cortical aerenchyma. The other integrated root phenotype which was equally effective in contributing to high shoot biomass under drought was comprised of high nodal root number at depth, small root cross-sectional area, and small metaxylem vessels. Therefore, it may be most practical to focus QTL mapping on the composite traits and to dissect the related physiological mechanisms once a QTL is identified.

A common discussion amongst screening protocols is whether screening should be done under controlled conditions, or under natural field conditions—both of which have strengths and weaknesses (Plessis 2023; Juenger and Verslues 2023; Vadez et al. 2024). Field conditions represent critical relevance, but they are often unreliable (i.e. the target conditions do not occur every season); stresses are not uniform over plots and between seasons, screening is limited by seasonal activity and hence slow, and results from one field might not correlate with those from another field. Disease screening is limited to pathotypes present in those specific locations tested. Controlled environments have natural advantages in precision, allowing on-demand, potentially year-round screening (and hence allow much faster progress), screening can be carried out at varying stress levels, timing of incidence, across a broad range of pathotypes, and lower costs. However, the precision of these conditions means eliminating many other environmental factors that might also contribute to the trait of interest. For example, controlled environments typically restrict the volume of the root zone and thus the total amount of resources available for uptake, typically expose the plants to lower light levels than under field conditions, often involve substrates very different from field soils, and result in unavoidable differences in soil hydraulic conditions (Passioura 2006).

To achieve the best outcomes of trait development, an effective integration of both types of screening is required. Controlled environment screening lends itself to initial discovery efforts due to its higher precision of results and on-demand nature. This enables screening of larger panels of material without requiring impractical levels of replication. However, the controlled screening protocols need to be validated for their phenotypic representation of field performance. Hence the workflow would be to first establish correlations between controlled environment and field screens. The controlled environment screen is the basis for discovery efforts, being applied to GWAS, biparental mapping activities, and initial validation of introgression products (stages 3, 4). Shortlisted germplasm is then validated in the field. Final validation of introgression products (stages 5, 6) then moves back to multi-location field evaluation to confirm effectiveness of the QTL in elite germplasm.

Screening protocols are developed for application to the target material, both potential diverse donors and elite breeding material. Here, “elite material” is defined as breeding lines performing equivalent to the current cohorts of material under early or advanced yield trials in the breeding programs. Elite material does not include landraces or very old (15 + years) varieties. There is always some level of variation in elite material, and if this is sufficient to meet trait targets it is possible to short-cut some trait development activities. For example high-performing elite material would be the preferred donor, and so QTL mapping could be focused on Mendelizing the trait and simplifying selection through enabling reliable marker-assisted selection—this still has substantial value even if the locus is found in elite lines. Once identified, however, stage 4 activities would be minimal since the gene is already in elite backgrounds, and those elite lines could be used directly by breeders –together with the appropriate marker system. Alternatively although some elite lines may perform better than others, none may reach the level of improvement required. In this case the elite variation gives a target that prospective landrace donors must exceed; they are less likely to contribute if they cannot beat current elite lines.

Besides evaluating the elite material, the screening protocol can also be applied to identifying new donor material, such as landraces that have substantially better trait value than seen in elite material. Screening for new donors could be optimized using the Focused Identification of Germplasm Strategy (FIGS) to enrich the pool of potential donors (Street et al. 2016), for example by screening Genebank accessions that were collected in regions where the target stress frequently occurs. It also lends itself naturally to GWAS investigation, and hence stage 2 may give the first indication of loci contributing to the trait. However GWAS should not be seen as the end of discovery, as outlined in stage 3. One factor showing promise for traits such as abiotic stress is to identify donors with diverse backgrounds – geographic origins, genetic relationship, or even physiological mechanisms if these are known. These diverse origins have a better chance of representing independent evolutionary origins for improved performance, and hence the possibility of complementary genetic mechanisms which could be combined to produce even greater performance, maximizing the output of trait development activities.

### Stage 3: locus discovery

Following GWAS, much more work is necessary before those research outputs can be used in breeding. Stage 3 focuses on deriving rigorous evidence for the genetics contributing to the trait. Donors identified in stage 2 are used to produce biparental mapping populations for QTL mapping. Donors may come from GWAS results (carrying the favorable haplotype) or may simply have a highly desirable phenotype. GWAS may indicate where a QTL is likely to be, however, it is biased towards common, large-effect loci (Bernardo 2016). Depending on the reference genome used, GWAS may not detect some important alleles as they may be absent in certain genetic backgrounds (Tranchant-Dubreuil et al. 2019). GWAS also gives no indication on whether a QTL is likely to work in elite backgrounds, which becomes more important as the divergence of the donor from elite material grows (Juma et al. 2021). Following identification of GWAS peaks, favorable haplotypes can be identified based on the phenotypic data, and the frequency of that favorable haplotype in the elite breeding pool can be assessed. GWAS thus requires significant follow-up work to address issues related to using a new locus in mainstream breeding.

For maximum benefit in breeding, a new locus identified through QTL mapping or GWAS must be:

- Able to confer significant improvement (effect size) in the trait of interest
- Effective in elite backgrounds (vs. in non-elite backgrounds)
- Stable across elite backgrounds
- Effective under field conditions

These criteria reveal two common tradeoffs of depth vs. breadth: evaluating stability across different backgrounds versus reducing false discovery through evaluating a large, rigorous population in one background; and secondly, evaluating large populations in one controlled environment vs. evaluating much smaller panels in numerous locations/environments. The primary expenses in locus discovery efforts are typically time (to produce and screen populations) and genotyping costs, which have driven many efforts to “optimize” trait development and may lead to compromises in data quality and reliability.

For example, researchers often phenotype/genotype early generation, non-fixed (e.g. F_2_) populations to speed up the discovery process. However, F_2_ populations preclude replication at the level of genotype; they cannot be re-examined in multiple experiments or environments, and replication is only possible by essentially replicating the entire population, with associated increases in genotyping costs. Thus minimizing time (while holding genotyping cost constant) sacrifices repeatability and rigor. This may be offset by increasing population sizes, but to do this rigorously then entails substantially larger genotyping costs.

Likewise in an attempt to reduce genotyping costs (Arbelaez et al. 2019), many researchers attempt some form of bulked segregant analysis by genotyping just extreme or selected individuals. However, this dramatically reduces population size and leads to overestimation of effect sizes via the Beavis effect (Xu 2003; King and Long 2017), increasing the potential for false positive discovery, and resulting in larger confidence intervals. While such methods may work in ideal situations with single genes of very large effect (such as mutant populations or some disease resistance cases), they are significantly underpowered for investigations involving multiple loci, loci with modest (but still useful) effect, or traits with high environmental variability. This reduction in power raises significant issues of false-positive outcomes, posing significant problems in application of such QTLs in mainstream breeding.

Although evaluation will always be limited by resource constraints, all four criteria outlined above must be addressed to have confidence in using a new locus. Addressing these criteria can be kick-started by properly planning population types used in discovery/confirmation. Essential considerations for biparental population construction include:

- Use of relevant, elite recipients. Ideally a modest number (3–5) of elite lines from target breeding programs must be identified which will act as recipient parents in population construction.
- Throughput of screening protocols. If accurate, high-throughput screening protocols are available, different population types may be targeted such as F_2_-derived RILs, compared to situations where screening can only handle a few lines at a time, where chromosome segment substitution lines (CSSLs) may be more appropriate.
- Replication of genotypic effect: early discovery efforts are likely to have high levels of noise from the donor genome, and the time required to produce fixed populations (e.g. F_4_) to enable proper, replicated and repeated trials is usually well spent.
- Appropriate population sizes, considering the mapping strategy.
- Appropriate genetic structure of populations. In most cases this means populations must be genetically random (as in RILs etc.) or systematic (CSSLs), but it is critical the populations are not pre-selected in any way, especially for the trait of interest.

Some common population types are outlined in Table 1, along with their advantages and disadvantages. In many respects the choice of population type will depend on factors such as the throughput of screening protocols, but it is worth noting some features of chromosome segment substitution line (CSSL) populations. CSSL populations have historically been used only rarely but they present a number of advantages. They are fixed populations, allowing unlimited replication and repetition of experiments. Combined with their relatively high recovery of the elite genome, this allows confirmation of phenotypic effect immediately after discovery, and concurrently with many other aspects of trait development (stage 4), giving substantial benefits for confidence and saving time. The higher recovery rates of the elite genome allow more confident inference of QTL effect in an elite background (compared to an F_2_-derived RIL which is still ~ 50% landrace), and importantly, gives a direct estimate of the absolute effect size of the locus. The required screening throughput required is typically much smaller, as the “mapping” strategy in CSSL populations is fundamentally different due to this high recipient parent recovery rate. In an F_2_-derived population each line is compared fundamentally against all other lines to help disambiguate genotypic signal of a QTL from background noise, necessitating a large population size (> 200 is a common minimum) to be assessed simultaneously to control background genotypic noise. In a largely pure background such as a CSSL, the fundamental comparison is whether a given line is better than the recurrent parent and thus the screening could be limited to one line at a time, compared to the recurrent parent. This can be exceptionally useful for traits with very low throughput, expensive, time-consuming phenotyping protocols. The high recipient parent recovery rate of a CSSL greatly reduces the time and expense of deployment (stage 4); once a locus is identified, most of the background cleanup is done, and all that remains is recombinant selection to remove linkage drag. Finally, CSSL populations can typically cover the donor genome in as few as 40–60 lines. This both enables screening more populations (or permits lower-throughput screens), and drastically reduces the cost of preserving the population. CSSL populations can then become value-added resources for future discovery efforts – if a new GWAS study identifies a favorable haplotype, this may well be captured in an existing population, thereby saving two years of work and thousands of dollars to validate QTLs for other traits of interest.

**Table 1:**
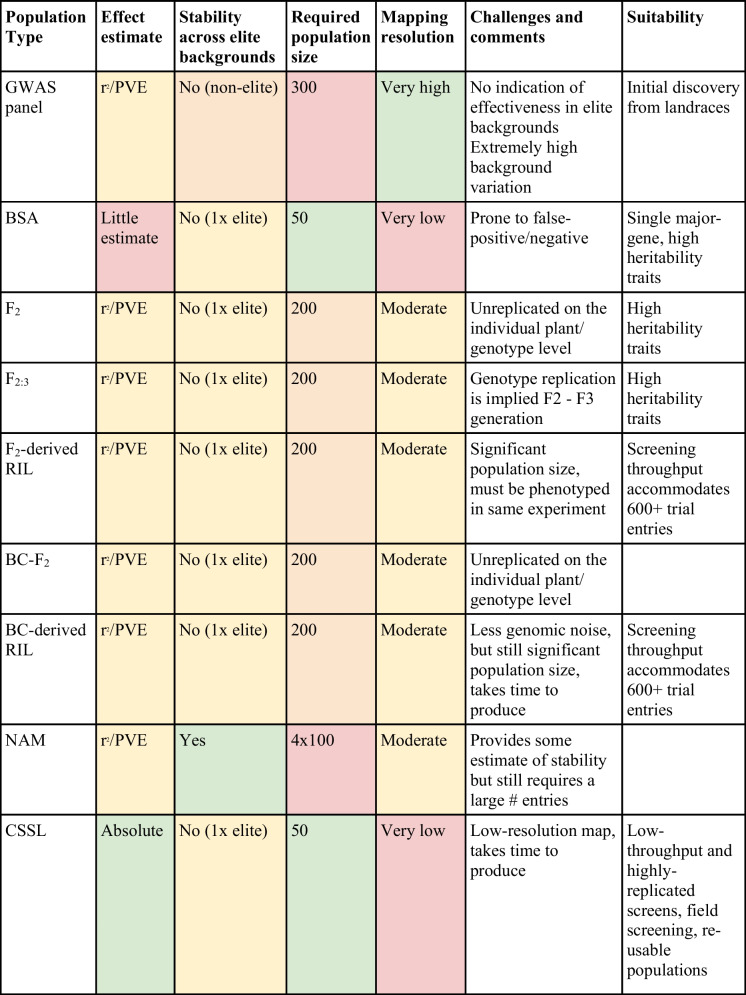
Common types of discovery populations^1^, with strengths, weaknesses and requirements for success. The required population size indicates the number of lines or individuals required in the described populations

^1^GWAS: Genome-wide association study, BSA: Bulked segregant analysis, RIL: Recombinant inbred line, NAM: Nested association mapping, CSSL: Chromosome segment substitution line, PVE: Percentage variation explained

#### QTL effect size

Determination of QTL effect size is one of the most important parameters in determining the value of a QTL, and it is typically approximated by the percentage variance explained (PVE, r^2^) determined by biparental mapping software or the regression coefficients from GWAS. Other measures of effect that do not depend on the frequencies are the additive effect, defined as half the difference between genotypic values of two homozygotes at the locus, and the average effect of allele substitution, which is measured by regression coefficient. However the PVE in the original study population is not always a great predictor of effect size in a different population, and hence is not a reliable measure of value (Bernardo 2020) – the reasons may include differing allele frequencies between the populations and differences in the additive effect of the locus across different backgrounds, amongst others. A QTL’s effect may be swamped by another QTL of huge effect in the study population (and likely to be estimated less precisely) – however if the larger QTL is fixed (monomorphic) in elite material, the smaller QTL may still hold significant value in pushing the trait value above what is currently found in elite material. Likewise the PVE is specific to the population and screen assessed. Hence rather than PVE, trait development needs to aim for a measure of absolute improvement conferred (i.e. additive effect, the difference of genotypic values of homozygotes) in the backgrounds present in target populations. The additive effect is approximated by average effect of allele substitution in the inbred case (Falconer and MacKay 1996). This may be estimated from biparental mapping populations or GWAS analyses (estimates are for example available in output files from QTL ICIMapping, Meng et al. 2015), but this will require rigorous population sizes and statistical design of experiments to minimize the Beavis effect (Xu 2003) and achieve any accuracy. It is simple to estimate absolute benefit from CSSL populations, since these are already in a mostly-elite background and can be compared directly with the original recipient line. Disease resistance loci require a special consideration; rather than PVE or even absolute effect size, the spectrum of resistance must be the unit of evaluation. This information will feed directly into designing a resistance strategy to promote durability.

Bringing the above considerations together, it may be possible to develop a general-purpose mapping strategy to provide a reasonable first evaluation of the QC parameters for locus discovery. This approach would involve generating several populations for discovery:

1. A substantial population (preferably CSSL) in the most important elite background. This should target 100% donor coverage and 85% + recovery of the elite background, which can be achieved with BC_2_-derived fixed lines. The CSSL lines will answer several critical questions: they are well suited to estimating absolute effect size, and once a high-value CSSL line is identified, this can be screened across multiple environments (field sites, experimental conditions, pathotypes) to give an initial evaluation of environmental stability. They may also form the donor lines used to generate supplemental biparental populations.
2. Several additional, perhaps smaller, biparental populations. These may follow CSSL approaches, or be F_2_ or BC-derived fixed lines, and hence suitable for a nested association mapping (NAM) approach. The primary purpose of these lines is to provide some evidence of stable QTL effect across elite backgrounds, but they could also be used in a NAM approach to help narrow the QTL interval.

This approach may be more expensive than creating a single population but these populations can then be used repeatedly for rigorous, multi-location and multi-treatment trials to pin down the potential value of a new locus.

### Stage 4: deployment

If promising loci are identified in stage 3, the next step is to produce more rigorous genetic materials as a prelude to introducing these loci into mainstream breeding. The focus of stage 4 is on producing two major items:

1. Reliable marker systems.
2. High-quality elite lines containing the new locus.

At least some diversity of donor and non-donor material is now known from stage 2 and 3 results, and this now is easily supplemented with resequencing data (McNally and Henry 2023). Together these are a powerful combination for designing accurate marker systems, even for large QTL intervals, with accuracy metrics can be easily determined (Platten et al. 2019). These markers will be used both in the introgression process, and eventually as production markers in mainstream breeding. Of note are the peak markers, which must be as accurate as possible for the target QTL, preferably across a wide diversity of genetic backgrounds and targeting the allele that represents the derived state (i.e. most recently arisen in evolutionary history, Platten et al. 2019). Flanking markers for recombinant selection are better to target the primary recipient variety that will be used for introgression; the upgraded recipient will be used as the donor to introduce the gene to multiple elite backgrounds in stage 6, so these flanking markers could be re-used later for further recombinant selection into additional elite haplotypes.

The creation of high-quality elite lines containing the new locus is the first step in introduction to the mainstream breeding program. To achieve this, the following quality criteria must be considered:

- Recipient: must be highly elite and relevant to target breeding programs.
- Introgression size: typically recombinants are targeted < 1 cM from the target gene (or flanking a QTL interval), for a total introgression size of < 2 cM to reduce the likelihood of linkage drag.
- Purity: recipient parent recovery rates > 95% is a typical target; this may be higher for undesirable backgrounds such as wild species.
- Recovery of key genes from recipient.
- Creation of coupled linkages.

If the recipients for stage 4 are the same as used in discovery population development, positive discovery lines could be used as donors to short-cut the deployment process; CSSL lines are particularly suited to this workflow, and deployment could be as few as three generations of recombinant selection and fixation. Since deployment is not well-suited to high-throughput situations, it would normally be focused on one, or a few, specific recipients rather than large numbers; the focus is quality not quantity. Any line passing these criteria is going to be a near-isogenic line in the nominated elite background(s). The NILs thus produced are then excellent for penultimate stages of validation in stage 5.

In the event that no major loci could be identified as contributing significant improvement in a particular trait, phenotypic introgression could be appropriate (Bernardo 2009; Gorjanc et al. 2016; Allier et al. 2019; Han et al. 2017). This would involve multiple breeding cycles of balancing selection for trait improvement and yield, until progeny can be produced having higher trait value but comparable yield performance. This process is longer and more expensive than marker-assisted introgression and loses some of the value of trait development but may be necessary in certain situations – though its use has been poorly documented in rice.

### Stage 5: validation

By the time materials are ready to enter stage 5, high quality near-isogenic introgression lines will be available in a fully-elite background. Stage 5 is focused on detailed phenotypic validation and characterization of the elite NILs, to show effectiveness in the elite background and target environments. Key activities include:

- Evaluation of elite near-isogenic donor lines for effectiveness under screening protocols developed in stage 2. This could include expanding the characterization to a wider range of conditions, in a range of stress severities, against additional isolates, and at other locations.
- Evaluation of elite lines under field conditions, such as hotspot locations, and evaluation of potential penalties for yield and agronomic performance traits.
- Characterization of off-target (pleiotropic) effects. This is important for yield as well as basic agronomic performance traits (plant height, crop duration, etc.) and interactions with related traits – e.g., determining whether drought genes have a penalty (or advantage) for salinity tolerance.

Most of these activities would be carried out by phenotyping teams or breeders, but stage 5 also presents an opportunity to refine the genetics. If there is a need to reduce the confidence interval, selection of recombinants in the elite NIL background allows for powerful and sensitive tests. In parallel, more detailed characterization of physiological mechanisms and cloning of QTLs (transgenic validation; knockout, overexpression etc.) are feasible at this point. These are valuable (though not required) in providing definitive proof of a gene’s involvement in the trait of interest, increasing the accuracy of marker design and profiling of presence/absence in new germplasm.

### Stage 6: scaling – line augmentation

At this stage, materials are already available that enable the use of a new locus in mainstream breeding. Elite NILs from deployment can be made available, along with marker systems to allow selection, and information to support the usefulness of the new locus. However there are two disadvantages related to NILs produced in deployment:

- The recipient background is inevitably from an earlier breeding cycle, and may represent a compromise amongst the target breeding programs/pipelines.
- It is not desirable to make extensive crosses with a single background, which would result in rapid depletion of genetic variance in the breeding pool.

To counter these factors, the availability of a new locus needs to be scaled up to multiple elite backgrounds, hence line augmentation is focused on rapid introgression to multiple backgrounds. Since the donor material is a NIL in an elite background, concerns about purity and quality of products are not so prominent. Simulations have shown the augmentation process is better to focus on a single backcross, with progeny selected at the BC_1_F_1_ and BC_1_F_2_ stages first by presence of the target genes, and secondly by the estimated breeding value based on genome-wide genotype (GEBV; Platten and Fritsche-Neto 2023). The resulting products maximize breeding value (rather than recovery of a particular parental genome), and are more useful as parents in subsequent breeding cycles. Once completed, augmentation products are then phenotyped using standard protocols to verify their effectiveness, and materials communicated to breeding programs.

By the end of stage 6, breeders have access to the following:

- Effective, relevant screening protocols,
- Well-validated loci, proven in multiple elite backgrounds and across multiple environments,
- Reliable marker systems to select for these loci using high-throughput, low-cost genotyping platforms,
- Elite donor material in multiple genetic backgrounds to use as parents in the crossing program.

This therefore addresses the primary needs of breeders to confidently adopt pre-breeding products. Although such a detailed process involves additional time and cost, this protocol will facilitate continuation of the work after discovery (stage 3), to improve uptake and impact of pre-breeding products. Much of this work can be centralized (achieving economies of scale, e.g. deployment and augmentation) or leveraged off existing activities (field validation, conducted alongside existing breeding trials). This requires a close collaboration between the trait development teams and breeding and seed systems teams, but would allow substantial extension of results for less expense, and also helps to communicate these results to the downstream teams.

## Application of TDP outputs in breeding programs

The outputs of trait development can be most effectively applied in breeding activities and can be utilized in breeding at multiple points:

- Validation data from stage 5 can be used to inform areas of optimal impact (geographic areas, and environmental conditions), allowing breeders to design a selection target;
- Elite donor material from stages 4 and 6 of the TDP can be used as parents to introduce new genes to breeding populations;
- Marker systems from stage 4 can be used to fix target genes in breeding populations;
- Screening protocols can be used to confirm improved performance for the target trait

Major-gene selection is typically integrated into the early (e.g. line fixation) stages and will require appropriate planning of population sizes, and consistent application of selection pressure for target genes.

Required population sizes depend on several factors: generation at which selection is applied and the selection target in terms of fixation (both of which influence the expected segregation ratio), the number of loci to be fixed, the required number of positive segregants identified (*R*), and the acceptable failure rate of not finding *R* positive segregants (*F*). This can be calculated as outlined in Cobb et al. (2019), notably in Table 1, which indicates that it is difficult to handle more than 2–3 major genes through determinate MAS with reasonable population sizes. The efficiency of this process can be improved by implementing a 2-stage MAS selection, for example at the F_2_ and F_4_ generations (Table 2) by eliminating any segregant negative for any of the target loci in the F_2_ generation to enrich the target loci. Since one locus (any one of the target loci) is fixed-positive, there is no benefit in retaining fully-heterozygous progeny, as these are functionally no better than the F_1_ generation that precedes it, and are highly unlikely to contribute fully-fixed segregants in the final fixation.

**Table 2:**
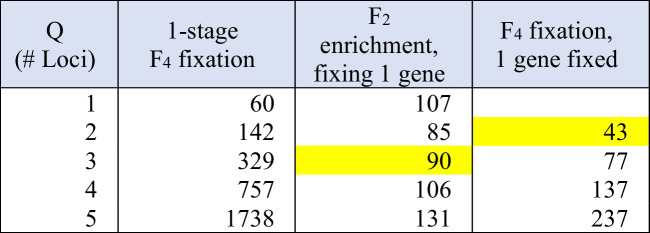
Population sizes required (number of lines) for fixing variable numbers of genes under 1- or 2-stage MAS

Population sizes assume *R* = 20 positive segregants and *F* = 5% acceptable failure rate

Segregation ratios (P) are given by:

1-stage F4: 0.4375^Q

2-stage F2 enrichment: 0.75^Q – 0.5^Q

2-stage F4 fixation: 0.5833^(Q-1)

Besides population sizes, consistency in genes targeted for MAS is also important. Positive segregants must be recycled as parents for determinate fixation of a gene via MAS. Some incorporation of major gene value needs to be included in parental selection algorithms. For consistent improvement in the breeding pool, the same gene must be consistently selected in all populations where it is segregating; inconsistent selection will lead to greater stochastic variation in prospective parents, and hence greater drift. This effect will be magnified if the target gene is otherwise very rare in the breeding pool, as would be expected for most loci coming out of trait development. A consistent prioritization of genes is required *across* families in a breeding program: each gene is assigned a priority by the breeder, taking into account the need for the trait, and the value of the gene for improving that trait. In any given population, the 2 or 3 highest-priority loci that are segregating are targeted for selection, ensuring a consistent application of selection pressure, eventually driving the favorable allele to fixation. Fixation then frees up additional selection pressure for the next priority gene.

## Trait development pipeline case studies in root biology

**Fig. 2:**
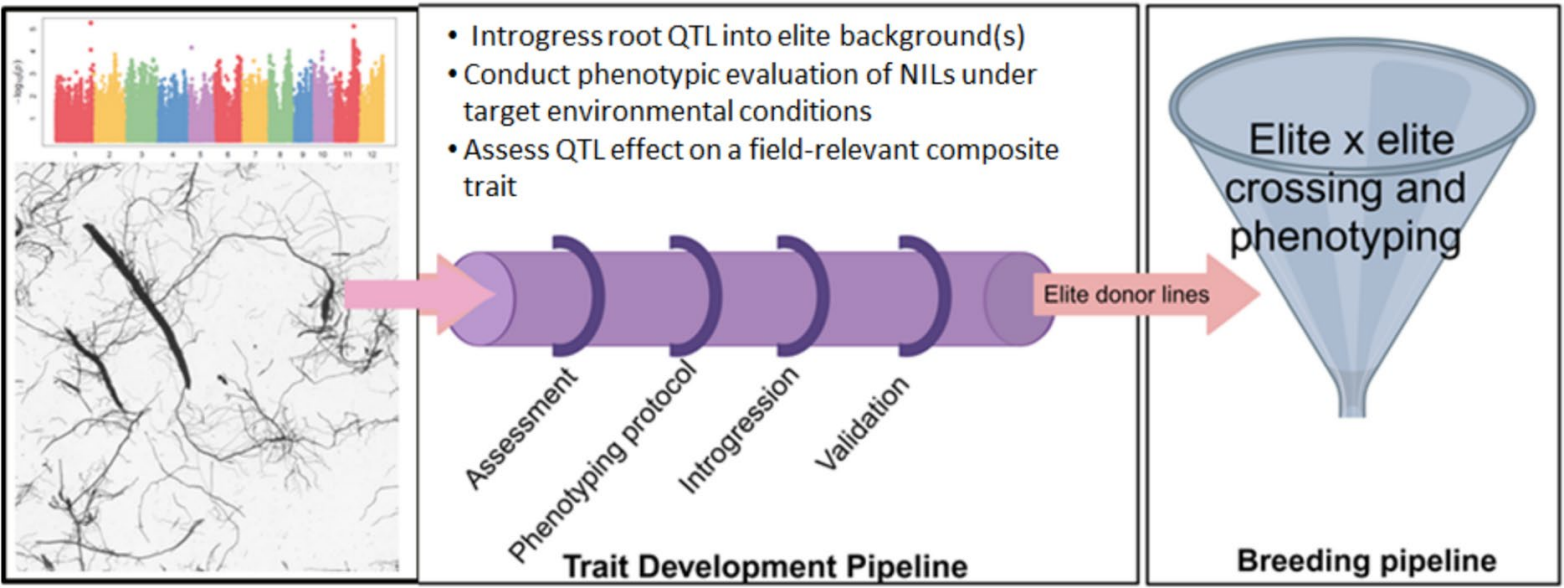
A potential trait development strategy for identified root traits and QTLs. The majority of root biology studies have focused on the early stages of the Trait Development Pipeline, typically ending with locus discovery. An increased effort to link these root biology studies with breeding pipelines is needed, especially in terms of introgression into elite backgrounds and validation under target environmental conditions

In terms of potential for contribution to breeding via the Trait Development Pipeline, the field of root biology stands out for its prolific efforts to identify QTLs related to specific traits that have been proposed to affect crop yield. In the case of rice, more than 900 root-related QTLs have been reported (Courtois et al. 2009; Daryani et al. 2022). Unfortunately, to date only very few of these identified root QTLs have been actively taken up by breeding programs. Birsa Vikas Dhan 111 was developed by introgressing root QTLs from Azucena into the background of Kalinga III and released as a variety for drought-prone areas in India (Steele et al. 2013). DRR dhan 60, DRR Dhan 66, and WGL 1487 were released in India (Anila et al. 2018; Mahadevaswamy et al. 2020), and FyVary32 was released in Madagascar (M. Wissuwa, personal communication)—all of which were developed by introgression of the *Pup1* QTL for low phosphorus tolerance into the background of popular varieties. *Pup1* was found to be due to a protein kinase, Pstol1, that promotes early root growth in tolerant lines, which in turn facilitates greater phosphorous uptake (Gamuyao et al. 2012). Of these varieties with *Pup1* introgressed, DRR dhan 60 appears to be the most resilient and widely adopted thus far with yields recorded at 4.8 t ha^−1^ under 50% application of phosphorus and 5.5 t ha^−1^ under 100% phosphorus application, and has recently been requested by the government of Bangladesh for immediate release in their country (R.M. Sundaram and Anantha M.S., personal communication). One of the most extensively-characterized rice root QTLs, *dro1* (Uga et al. 2013) promoting a deeper root angle, has thus far mainly been restricted to use in the IR64 background, since the favorable allele is already present in many other genetic backgrounds of current varieties and breeding lines (e.g. Pandit et al. 2020; Singh et al. 2021). However, a set of multiple root QTLs including *dro1* have recently been reported to confer yield advantage under drought in the background of Colombian variety FEDEARROZ 60 (Deshmukh et al. 2024). In reference to the Trait Development Pipeline, most of the rice root work to date has focused heavily on development of phenotyping protocols (which requires deep understanding of which component root traits are most critical for the desired composite phenotype, e.g. yield or biomass under stress; Kuijken et al. 2015), and locus identification. More efforts on the deployment, validation, and scaling stages of the Trait Development Pipeline are necessary for more research outputs from root biology to move forward in breeding pipelines (Fig. 2). Here we provide several examples of root biology studies from IRRI, and considerations to guide researchers in how those studies could be further advanced in the context of the Trait Development Pipeline.

The first example was a study to identify potential QTLs for seedling stage drought tolerance under dry-direct seeding that could be contributed by traditional variety Aus 276. Two bi-parental mapping populations were evaluated over two field seasons (Sandhu et al. 2015). Of note were QTLs co-locating for root hair density and nutrient uptake (shown in Sandhu et al. 2015 Fig. 5), which were concluded to be a main result from this study that could contribute to breeding. With this output as a starting point and based on the Trait Development Pipeline principles outlined above, the QTL of interest would require further validation due to the relatively low LOD score (~ 4.0), the presence of a potentially faulty marker (as indicated by the steep dropoff in LOD score between the 2 QTLs), and the additive effect values which do not clearly indicate that the locus was contributed by Aus 276. As next steps, it is recommended to repeat the phenotyping and QTL analysis. If it will be necessary to regenerate a population, it is advisable to use an elite/more recently developed background rather than IR64 or MTU 1010 as previously used, as these are no longer competitive with the current elite material and one key aim of the Trait Development Pipeline is to produce such “elite donor lines”.

In a second example, Siangliw et al. (2022) evaluated a Southeast Asian panel that had been selected from the 3,000 rice genomes (3 K RG; Wang et al. 2018) with the aim to identify drought tolerance donors that might already possess some adaptation to conditions in Southeast Asia. The panel was grown under two field seasons, with root crown, root anatomy and root architecture traits characterized under both transplanted lowland and direct seeded conditions and GWAS conducted on all traits. In this study, biomass under drought was correlated with yield under drought, as well as high crown root number and smaller stele and metaxylem vessels. GWAS co-locations between seasons were identified, as well as candidate genes and favorable alleles in terms of SNP effect from among the three most promising co-locating loci. Subsequent to this publication, it was determined that the same six accessions were the donors of the favorable alleles for all three loci. To further advance the favorable alleles identified by Siangliw et al. (2022) via the Trait Development Pipeline, it will be necessary to address concerns about the low significance level of the GWAS peaks and the consideration of peaks represented by LD outliers, the probability that the phenotypic effects were not likely due to single SNPs, and the possibility of population structure as suggested by the same donors being listed for all three candidate loci. It is recommended that since there is high confidence in the relevant component root traits contributing to biomass under drought in this study, the panel could be re-phenotyped under more controlled conditions to reduce the error as compared to the results generated in the field, with the aim to improve the significance level of the GWAS peaks. If the GWAS loci appear promising/reproducible, further confirmation may be derived from biparental populations. This would also be a first step to demonstrating these QTLs are effective in elite genomic backgrounds. It is also recommended to check the frequency of the haplotypes in the elite breeding pool. Those favorable haplotypes that are rare in the current elite breeding pool would be prioritized for introgression into an elite background in stage 4.

A final rice root biology example is an extensive field yield/root architecture screening (Liao et al. 2022) of a panel of accessions from the aus subgroup, which was expected to provide promising sources for breeding since it originated in stress-prone regions. Following characterization of about 250 aus accessions over four field seasons, the yield stability was determined to be correlated with stable deep root growth. A path analysis including the root dry weight at depth results from the field together with root length and diameter class results from a greenhouse lysimeter study, root number and angle results from a seedling stage basket study, and root depth-related herbicide damage score from an herbicide-at-depth experiment indicated that it was the nodal root diameter class that linked vegetative stage with reproductive stage root traits. GWAS conducted for grain yield and each root trait yielded very few significant peaks; therefore the approach of identifying multi-trait co-locations of non-significant peaks (-log_10_(P) = 3, window of 1 kb) between root traits and grain yield was used while demonstrating the low likelihood of false positives. Consistent with the phenotypic correlations, nodal root diameter class showed the most co-locations with grain yield under drought. Favorable haplotypes from among the co-locating loci were identified based on the proportion of accessions with high grain yield under drought per haplotype. Following this publication, donor accessions with the favorable haplotypes were listed (which ranged from one to nine accessions), and the frequency of the favorable haplotypes in the current elite breeding pool was determined (some were rare, some were present in ~ 30% of the elite lines). Suggestions about these results in the context of the Trait Development Pipeline are that it is preferable to see a significant GWAS peak—there are concerns whether the identified loci are real, and that it would be good to narrow down the window size of the favorable haplotypes. To confirm the QTLs identified, a biparental population segregating for the favorable haplotype could be screened, which may already be available in the collection of trait deployment populations at IRRI. In addition, elite breeding lines with rare favorable haplotypes might be prioritized for crossing.

In summary, while these examples present considerable advances in characterizing the diversity in rice root response to drought, there is still a lot of work required to validate the identified loci before an elite donor to be used in breeding is developed. Between the inherent heterogeneity of the soil in which roots are growing and the variable nature of root growth, root biology studies tend to have a high degree of phenotypic noise which may affect the ability to detect reliable QTLs for important root traits. Approaches to reduce the degree of phenotypic noise include the use of more controlled environments as complementary studies, evaluation of candidate loci in cleaner genetic material (i.e. NILs as opposed to landraces), and increasing the number of experimental replicates.

The recommendations in the context of the Trait Development Pipeline point to the potential value of using controlled environment conditions to validate target component trait results, which may be of interest in better linking upstream and applied programs which typically flow only in the direction of controlled environment to field. Phenotypic and genetic characterization of the breeding pool (or at least existing varieties) is a critical part of the Trait Development Pipeline, in order to know which gaps should be filled by the trait development work. The observed limitation in progression through validation to application is not restricted to root biology: surveys across most traits show a bottleneck in validation/deployment between discovery (stages 2 and 3) and application (stage 6). It is recommended that the populations for QTL discovery should involve at least one elite parent, typically the recipient, so that those introgression lines can directly demonstrate the value of a new QTL and serve directly as an elite donor in breeding. Finally, given the vast number of rice root QTLs that have been identified and reported in the literature, there will have to be some prioritization of what to move forward into the Trait Development Pipeline from these and other studies. At IRRI, validation by screening of existing populations that are segregating for the favorable haplotypes that are rare or absent in the elite breeding pool will be prioritized, to avoid the need to generate new populations.

## Ways for upstream researchers to contribute to the TDP

Part of the bottleneck in validation/deployment may be due to the resources required in meeting these objectives (time, space, availability of current elite material, field screening capacity). Meeting breeder’s requirements is likely to require a collaborative approach; research groups with better capacity in upstream areas (mapping, bioinformatics, cloning) will need to partner with others working more downstream (field screening, population development, genetic studies) to deliver on the required outputs. The Trait Development Pipeline provides a framework to guide this teamwork in order to take advantage of the value of diverse specialists (geneticists, pathologists, physiologists, grain quality scientists, etc.), all the while guiding these contributions by the outputs required for impact in breeding. This in turn allows breeders to focus on making the right crosses, multi-environment testing, and linking with national testing systems to get varieties released. Together this produces higher-quality outputs from both upstream and downstream activities.

The Trait Development Pipeline facilitates movement of information both upstream and downstream. While identified QTLs are an important milestone in trait development, they can also be starting points for further research such as validation of candidate genes, expression studies, and advanced physiological investigations. Candidate genes can be cloned and validated, and their effectiveness on the target phenotype in different genetic backgrounds can be assessed and further characterized. If a gene is found to be *ineffective* in some genetic backgrounds, upstream researchers could investigate what causes this unfavorable epistasis, which would further improve the predictability of breeding. In the case of root traits, their distribution in the elite breeding pool (or target variety) can be assessed to define phenotypes or associated haplotypes that are likely to fill gaps in the breeding pool. In the case of rare root traits/genes/QTLs, these can be validated in an elite background. In summary, there are many ways that upstream research can contribute to the Trait Development Pipeline, and a number of stages in the Trait Development Pipeline offer opportunities for basic science questions to be addressed. Finally, the multiple iterations of testing and refinement inherent in the TDP go a long way towards addressing the reproducibility crisis that has hit many areas of biology (Baker 2016).

## Conclusions

The Trait Development Pipeline is designed to maximize the likelihood of uptake and impact of upstream genetic research in mainstream breeding programs. By understanding breeders’ needs and concerns, the pipeline is specifically designed to explicitly address these factors and produce the knowledge and materials to make it as easy as possible to use these in the breeding process. Although it is designed primarily for rice with its rich store of large-effect loci, the same principles (and many of the same activities) apply to other species; it should be directly applicable to any species possessing active elite breeding programs and where useful naturally-occurring variation exists outside these programs. The principles can also be adapted easily to other situations such as de novo variation created by genome editing or mutagenesis. As such, the Trait Development Pipeline is a dynamic framework and will change as breeding progresses.

Given the large number of root traits and QTLs that have been reported in the literature as potentially useful for breeding, advancement of these research outputs in the Trait Development Pipeline is urgently needed. Prioritization should be given to those traits/genes/QTLs that are rare in the elite breeding pool (which necessitates genotyping the elite pool and assessing allele frequencies of major loci), that address the most urgent needs of the breeding program (i.e. in terms of stress tolerance or adaptation), and that can be quickly validated in an elite background. This process can be facilitated by aligning new discovery efforts (such as biparental populations) to the principles described in the Trait Development Pipeline.

Despite all efforts to make them attractive, it is still necessary for breeding programs to adopt the materials produced. However further steps are needed: results need to be communicated to target breeding programs. Online repositories of curated data will be exceptionally helpful in achieving this, but ultimately a close working relationship is needed between the upstream pre-breeding teams and mainstream breeding programs. Frequent two-way communication from up- and downstream work and an openness to improving the current status quo are needed on both sides, but if this can be achieved both sides stand to benefit from more sustained and rigorous application of trait development products in crop improvement.

## Supplementary Information

Below is the link to the electronic supplementary material.

Supplementary file1 (DOCX 26 KB)

## Abbreviations

TDP: Trait Development Pipeline
CSSL: Chromosome segment substitution line
NIL: Near-isogenic line
