# Supplementary material for: The rice Trait Development Pipeline: a systematic framework guiding upstream research for impact in breeding, with examples from root biology
Source: Plant Soil. 2025 Mar 29;514(1):29–46. doi: 10.1007/s11104-025-07399-2 (PMC12540618; doi:10.1007/s11104-025-07399-2)
Supplement: Supplementary file 1 — Supplementary file1 (DOCX 26 KB) [file 11104_2025_7399_MOESM1_ESM.docx]

**Platten et al.**

**The rice Trait Development Pipeline: a systematic framework guiding upstream research for impact in breeding, with examples from root biology**

**Supplemental Files**

**Table S1.** Advancement criteria across the stages of the Trait Development Pipeline.

| Stage | Outcome | Development criterion | Guiding questions |
| --- | --- | --- | --- |
| 1 | Trait development needs and goals | Value to breeding justifies investment | Is the trait needed in the breeding programs/is it specified in product concepts? |
|  |  | Where trait is required: - Breeding program(s) | - Which breeding programs/which product concepts? |
|  |  | - Target germplasm | - In what germplasm is it required? (e.g. early/med/late maturity). This will help prioritise list of recipients for biparental mapping. |
|  |  | - Target geographies | - What target countries/regions? |
|  |  | Level of improvement required | What is the target trait value? |
|  | Literature review | Phenotyping protocols | Are there known protocols for phenotyping this trait? |
|  |  | Donor material, including availability | Are there known donors for this trait? |
|  |  | Known QTLs/genes: - Confidence of efficacy in breeding programs | Are there known QTLs for this trait? - Validated in relevant elite germplasm (indica)? |
|  |  | - Ability to identify genomic location/polymorphism | - Is it possible to identify the chromosomal region in reference genomes? |
|  |  | - Availability of donor germplasm | - Are validated QTL donors available? |
|  |  | - Frequency in elite material | - Can/has the frequency of the donor haplotype been assessed in elite breeding material? Is it rare/absent? |
|  | Trait development strategy | Strategy document in place - What TDP goals are already met |  |
|  |  | - What key knowledge gaps remain |  |
|  |  | - What stage of pipeline development will commence |  |
|  |  | - Strategies to fill gaps in knowledge |  |
| 2 | Phenotyping protocol | Relevant to how trait is assessed in breeding program. E.g. field performance. Component traits can only be considered if there is substantial validation work. | Is phenotyping protocol interrogating the actual trait breeders need to improve? If not, what is the level of correlation between the phenotyped trait and the breeder's trait? |
|  |  | Suitable for use on mapping population and breeding populations (200+ entries) If this is strictly impossible, trait mapping strategies must be adjusted based on capacity. E.g. CSSLs. | Can the phenotyping protocol handle an adequate number of entries for effective mapping/screening (min. 200 entries)? If not, what will the mapping strategy be? |
|  | Variation for trait in elite breeding pool assessed | Elite breeding lines (current OYT cohort, core panels) do not contain the necessary mean and/or significant SD for the trait. | What is the mean and distribution for the trait in current elite breeding cohorts? |
|  | Donors identified | Donors possess mean trait values >2SD above elite material (based on elite mean + SD) | Have donors been identified with significantly higher trait value than observed in the current elite material? |
|  |  | Association mapping results - Appropriate panel design and statistical analyses, appropriate # phenotypic replicates | Have association panels been screened? - Have these followed best practices for panel composition, experimental design and analysis? |
|  |  | - Haplotype frequencies in elite material assessed | - Have haplotype frequencies for any association peaks been assessed in elite material? Is the favourable haplotype rare/absent? |
| 3 | Mapping populations | Appropriate for confident mapping (based on criteria outlined above) | Have biparental mapping populations been developed based on best practices (germplasm handling, population size and structure, appropriate elite recipient parents)? |
|  | Phenotypic assessment | Appropriate replication | Have phenotyping experiments been carried out using appropriate experimental designs and analysis? |
|  |  | Narrow-sense heritability >xx% | What is the narrow sense heritability of the phenotyping experiments? |
|  | QTLs identified | Seen across populations derived from multiple diverse elite recipients | Has the same QTL interval(s) been identified across multiple populations involving multiple elite recipient parents? |
|  |  | Effect size (NIL [+]/[-]) > 15%, OR  QTL PVE >10% | What is the PVE of QTLs recommended for deployment? |
|  |  | Confidence interval <5cM | What is the confidence interval for QTLs recommended for deployment? |
| 4a | **Genetic introgression track** |  |  |
|  | Reliable marker systems | Accurate peak markers designed and tested - FPR+FNR assessed, preferably zero at least in elite material | What are the accuracy metrics for markers promoted for use as peak markers? |
|  |  | - Technical performance shows >95% call rate and clarity | What are the technical performance metrics for peak markers, particularly as implemented at approved service provider(s)? |
|  |  | Flanking markers suitable for recombinant selection to <1cM/250kb | Where are the flanking markers for recombinant selection relative to the peak region? Are they applicable across multiple elite backgrounds? |
|  |  | Frequency of favourable haplotype/allele in elite material <5% | What is the frequency of the favourable allele/haplotype in elite material? Is it rare/absent (<5%)? |
|  | Elite donor lines | Elite status of introgression lines established: - RPP >95% | What quality metrics describe the deployment introgression lines developed? - Recipient parent recovery rate? |
|  |  | - Introgression size <2cM (gene) or <2cM larger than QTL interval | - Introgression size (<2cM/500kb for a gene)? |
|  |  | - Known linkage drag broken | - Known linkage drag broken? |
|  |  | - 3kg seed produced for field evaluation | - Sufficient seed for field evaluation (nominally 3kg)? |
|  | Value-added genetic resources | Identified opportunities realised: - Coupled linkages | Have opportunities for value-added products been explored and realised? - Coupled linkages |
|  |  | - Pyramids | - Pyramids |
|  | Fine-mapping resources | Recombinant individuals within QTL interval identified and fixed for fine-mapping | If needed, have recombinant individuals within QTL intervals been identified and preserved for fine-mapping exercises? |
| 4b | **Phenotypic introgression track** |  |  |
|  | Elite donor lines | Donor performance: - >90% of elite material for trait of interest | For introgression lines selected phenotypically, is performance: - >90% of elite material for the trait of interest? |
|  |  | - >50% of elite material for yield | - >50% of elite material for yield, grain quality etc.? |
| 5 | Validation of trait of interest | Effect is clearly seen on trait of interest in NILs | Do elite NILs from deployment show significant improvement in the target trait of interest? |
|  |  | Pleiotropic effects characterised: - Agronomic performance traits (maturity, height, tillering, etc.) | Assessment of pleiotropic effects of NILs: - What is the effect on general agronomic performance traits (maturity, height, tillering etc.)? |
|  |  | - Interactions in pyramids and coupled linkages assessed | - Are there interactions (synergistic/antagonistic for the trait of interest, additional phenotypes for non-target traits) in pyramids and coupled linkages developed? |
|  |  | Yield penalties assessed, within tolerable limits | - Are yield penalties absent or within tolerable limits (these will depend on the value of the trait of interest)? |
|  | Line Augmentation | Recipients are relevant to target breeding programs | Articulate value of recipients: - Value to breeding programs - Divergence from Deployment recipient |
|  |  | Breeding value (preferably) or recipient recovery rates calculated for each selected plant | Report on QC metrics achieved |
|  |  | Fixed lines containing new genes available | How much seed is available for crossing block? |
|  | Refined genetics | Confidence intervals reduced to <2cM | Fine-mapping: - Are confidence intervals for target QTL(s) reduced to 2cM or less? |
|  |  | Fine-mapped donor available | - Is a fine-mapped donor (containing the reduced/minimal confidence interval) available? |
|  |  | Candidate gene/transgenic validation supports usefulness of marker system | - Do gene validation efforts (transgenic, CRISPR etc.) support the primary candidate gene and accuracy of the marker system? |
| 6 |  | Available in elite material for crossing block | Is the elite donor available in the crossing block? |
|  |  |  |  |
